# Prevalence and characteristics of extended-spectrum β-lactamase genes in *Escherichia coli* isolated from piglets with post-weaning diarrhea in Heilongjiang province, China

**DOI:** 10.3389/fmicb.2015.01103

**Published:** 2015-10-08

**Authors:** Guofeng Xu, Wei An, Hongdong Wang, Xiuying Zhang

**Affiliations:** ^1^Department of Basic Veterinary Science, College of Veterinary Medicine, Northeast Agricultural UniversityHarbin, China; ^2^Inspection and Quarantine Technical Center, Sichuan Entry-Exit Inspection and Quarantine BureauChengdu, China

**Keywords:** post-weaning diarrhea, ESBL, PMQR, integron, TEM-52

## Abstract

**Objectives:** The purpose of this study was to investigate the prevalence of extended spectrum β-lactamase (ESBL) genes in *Escherichia coli* isolated from post-weaning diarrhea (PWD) piglets in Heilongjiang province, China.

**Methods:** Of 458 *E. coli* isolated from 589 fecal samples from PWD piglets, a total of 198 isolates were confirmed as ESBL producers by the double-disk synergy test (DDST). Polymerase chain reaction (PCR) and sequencing were performed to identify genes for ESBL, plasmid-mediated quinolone resistance (PMQR), and integrons.

**Results:** Of the 198 isolates, *bla*_CTX−M_ and *bla*_TEM_ were detected in 191 and 149 isolates, respectively. Sequencing revealed that 10 *bla*_CTX−M_ subtypes were detected, and *bla*_CTX−M−14_ was the most prevalent, followed by *bla*_CTX−M−55_ and *bla*_CTX−M−65_. Of the 149 TEM-positive strains, four were *bla*_TEM−52_ and the rest were *bla*_TEM−1_. Among the 198 ESBL-positive isolates, 173 isolates were found to harbor at least one PMQR gene, with *oqxAB, qnrS, qnrB, qepA*, and *aac(6*′*)-Ib-cr* being detected alone or in combination in 125, 114, 26, 24, and 45 strains, respectively. One hundred and fifty-five ESBL-positive isolates were also positive for class I integron (*int1*), and eight different gene cassette arrays were confirmed in 110 isolates by restriction fragment length polymorphism (RFLP) and DNA sequencing analyses, with predominance of *dfrA17*-*aadA5, dfrA12*-*orfF*-*aadA2*, and *dfrA1*-*aadA1* arrays.

**Conclusion:** To the best of our knowledge, this is the first report of the *bla*_TEM−52_ gene in pig *E. coli* isolates in China and this is also the first description of the coexistence of the *qnrB, qnrS, aac(6*′*)-Ib-cr, qepA*, and *oqxAB* genes in one *E. coli* strain.

## Introduction

Colibacillosis, caused by pathogenic *Escherichia coli*, is one of the most significant diseases in the swine industry. Diarrhea is the most common symptom of colibacillosis in pig livestock, and it is classified into two types: neonatal diarrhea (ND), which occurs in sucking piglets, and post-weaning diarrhea (PWD), which usually occurs within 2 weeks of weaning (Luppi et al., 2015). In general, ND and PWD infections can be prevented effectively by passive colostral and lactogenic immunity obtained by vaccination of the sow (Truszczynski and Pejsak, 2014). When immunity treatment fails, the antimicrobials such as cephalosporins, fluroquinolones, and co-trimoxazole are one of effective measures for the treatment of diarrhea. Accompanying the wide use of these antimicrobials in treating piglets diarrhea over the past decades, resistant isolates, especially multidrug-resistant isolates have been observed (Seiffert et al., 2013). The emergence and spread of antimicrobial resistance poses a serious challenge in controlling diarrhea in swine production.

The major mechanisms conferring resistance to cephalosporins rely on the production of extended-spectrum β-lactamases (ESBLs; Perez et al., 2007). ESBLs are a group of enzymes mediating resistance to most β-lactams approved in human and veterinary medicine, including extended-spectrum cephalosporins and monobactams but excluding carbapenems and cephamycins. These enzymes are now widely distributed worldwide in Gram-negative bacteria, which mainly exist in Enterobacteriaceae, especially in *E. coli*. A large surveillance of antimicrobial resistance has shown that the detection rate of ESBLs has increased significantly not only in human clinical *E. coli* isolates (Jones et al., 2013), but also in *E. coli* isolates from food-producing animals and food products, which has been raising concern about their possible transmission through the food chain. ESBLs are categorized into three types, which are TEM, SHV, and CTX-M, while the CTX-M β-lactamases have been divided into five groups known as CTX-M-1, CTX-M-2, CTX-M-8, CTX-M-9, and CTX-M-25 which have become the most prevalent in the world (Bonnet, 2004). ESBL-encoding genes are usually located on plasmids which are highly mobile and can harbor resistance genes to several other unrelated classes of antimicrobials, such as fluroquinolones, and co-trimoxazole (Wang et al., 2014).

The emergence of plasmid-mediated quinolone resistance (PMQR) has been reported since 1998, indicating that quinolone resistance can also be acquired through horizontal gene transfer (Strahilevitz et al., 2009). To date, four known PMQR determinants have been identified include Qnr proteins, aminoglycoside acetyltransferase AAC(6′)-Ib-cr, and the quinolone efflux pumps proteins QepA and OqxAB (Ruiz et al., 2012). Although the PMQR determine relatively small increases in the MICs of quinolones, these changes are sufficient to facilitate the selection of mutants with higher levels of resistance. Integrons, which are capable of capturing, excising and expressing genes cassettes that encode determinants of antimicrobial-resistance, play important roles in the horizontal dissemination of antibiotic resistance genes in bacteria (Cambray et al., 2010). Class 1 integrons were widespread in ESBL-producing isolates and play an important role in multi-drug resistance (Chen et al., 2010). As a matter of growing concern, PMQR genes and integrons have often been found to co-exist on the same plasmid with genes encoding ESBLs and to be co-transferred to recipients. Food animals colonized with ESBL-positive *E. coli* have been considered as potential sources of resistant *E. coli* causing infection in the community. *E. coli* antimicrobial drug resistance is strongly related to phylogenetic grouping (Barguigua et al., 2013). It has been observed that *E. coli* strains fall into four main phylogenetic groups (A, B1, B2, and D). Virulent extraintestinal strains belong mainly to group B2 and, to a less extent, to group D, whereas most commensal strains belong to group A and B1.

In the past few years, a rapid emergence and dissemination of ESBL-positive *E. coli* isolates have been increasingly reported in food animals in different countries and have gained considerable attention worldwide (Smet et al., 2010). Although there are many studies describing the molecular characteristics of ESBL genes among healthy food-producing animals, but limited data are available for the distribution and characteristics of ESBL genes in *E. coli* isolated from diseased animals (Liu et al., 2013a). Therefore, the main purpose of this study was to investigate the prevalence of ESBL genes in *E. coli* isolates collected from PWD piglets in the Heilongjiang province during 2010–2013 and assess the relatedness of PMQR genes and integrons with ESBL genes within the same strain.

## Materials and methods

### Bacterial isolates

From May 2010 to August 2013, a total of 589 fecal samples from piglets (< 2 months) with PWD were collected from 21 pig farms located in different geographic areas of Heilongjiang province, in the Northeastern China (Table 1). These fecal samples were collected from individual piglets using a sterile swab that was placed into an Eppendorf tube and transported to the laboratory within 12 h. The samples brought to the laboratory were immediately inoculated on MacConkey agar, and five randomly selected colonies with typical *E. coli* morphology were selected from each sample. The bacterial strains were identified using classing biochemical methods and confirmed as *E. coli* using the API-20E Bacterial Identification System (bioMérieux, France). All confirmed *E. coli* isolates were stored in Luria-Bertani (LB) broth, containing 20% glycerol at −80°C for further studies.

**Table 1:** **Total number of fecal samples collected from piglets with post-weaning diarrhea on different farms in Heilongjiang province**.

**Farm location**	**Farm**	**No. of fecal samples from each farm**	**No. of *E. coli* isolates from each farm**	**No. of ESBL-producing *E. coli* from each farm**	**Year**
Harbin	F1/F2/F3/F4/F5	35/35/34/34/30	26/20/25/30/29	11/4/10/16/15	2010/2010/2012/2012/2013
Daqing	F6/F7/F8/F9	22/18/25/10	21/10/16/7	8/0/5/2	2011/2011/2012/2012
Mudanjiang	F10	13	10	7	2013
Jiamusi	F11/F12	28/30	25/27	12/11	2010/2012
Suihua	F13/F14/F15	33/32/30	26/29/24	9/17/16	2012/2013/2012
Yichun	F16/F17	28/28	24/20	10/8	2010/2013
Jixi	F18/F19	30/30	24/21	11/9	2012/2013
Hegang	F20/F21	33/31	23/21	8/9	2011/2013
Total	21	589	458	198	2010–2013

### Antimicrobial susceptibility testing and ESBL determination

The minimum inhibitory concentrations (MICs) of ampicillin, ceftiofur, cefotaxime, amikacin, gentamicin, tetracycline, chloramphenicol, florfenicol, nalidixic acid, ciprofloxacin, enrofloxacin, trimethoprim/sulfamethoxazole, and colistin were determined by the agar dilution method according to the guidelines of the Clinical and Laboratory Standards Institute (CLSI; Clinical Laboratory Standards Institute, 2012). The antimicrobial concentrations were from 0.06 to 256 μg/ml. *E. coli* ATCC25922 was used as the quality control strain. All *E. coli* isolates were screened for the production of ESBLs by the double-disk synergy test (DDST) using both cefotaxime and ceftazidime, alone and in combination with clavulanic acid as recommended by the CLSI. The DDST was considered positive when the inhibition zone produced by the combined effects of either ceftazidime or cefotaxime plus clavulanic acid was ≥5 mm larger than that produced by either ceftazidime or cefotaxime alone.

### Detection of ESBL and PMQR genes

Plasmid DNA of *E. coli* isolates was obtained with the AxyPrep Plasmid Miniprep Kit (Axygen Scientific, California) following the manufacturer's instructions. All ESBL-positive *E. coli* isolates were screened by polymerase chain reaction (PCR) for the following ESBL genes: *bla*_TEM_, *bla*_SHV_, *bla*_CTX−M−1_ group, *bla*_CTX−M−2_ group, *bla*_CTX−M−8_ group, *bla*_CTX−M−9_ group, and *bla*_CTX−M−25_ group (Luo et al., 2011). The ESBL-producing *E. coli* isolates were also screened for the presence of PMQR genes: *qnrA, qnrB, qnrC, qnrD, qnrS, qepA, oqxAB*, and *aac(6*′*)-Ib-cr* (Robicsek et al., 2005; Gay et al., 2006; Park et al., 2006; Cavaco et al., 2009; Wang et al., 2009). The primer sequences and corresponding annealing temperature used in the PCR reactions are listed in Table 2. PCR was performed in 25 μl mixture containing 2 μl template DNA, 1 μl of each primer (10 nmol/L), 12.5 μl 2 × PCRMix (Haigene, China), 8.5 μl ddH_2_O. All PCRs were done as follows: 5 min at 94°C; followed by 35 cycles of 30 s at 94°C, 30 s at annealing temperature, 45 s at 72°C; and 7 min at 72°C. All positive PCR products were sequenced using an ABI3730 sequencer (Applied Biosystems, Life Technologies, Foster City, CA, USA) and their sequences were compared with genes in the GenBank using the BLAST program (http://www.ncbi.nlm.nih.gov/BLAST/). All aac(6′)-Ib-positive isolates were further analyzed by direct sequencing of the purified PCR products to identify *aac(6*′*)-Ib-cr*.

**Table 2:** **PCR primers used in this study**.

**Target gene**	**Nucleotide sequence (5′–3′)**	**Annealing temperature (°C)**	**Product size (bp)**	**References**
*bla*_TEM_	F: CATTTCCGTGTCGCCCTTATTC R: CGTTCATCCATAGTTGCCTGAC	58	800	Luo et al., 2011
*bla*_SHV_	F: AGCCGCTTGAGCAAATTAAAC R: ATCCCGCAGATAAATCACCAC	58	713	Luo et al., 2011
*bla*_CTX−M−1_ group	F: TTAGGAARTGTGCCGCTGYA^a^ R: CGATATCGTTGGTGGTRCCAT^a^	55	688	Luo et al., 2011
*bla*_CTX−M−2_ group	F: CGTTAACGGCACGATGAC R: CGATATCGTTGGTGGTRCCAT^a^	55	404	Luo et al., 2011
*bla*_CTX−M−9_ group	F: TCAAGCCTGCCGATCTGGT R: TGATTCTCGCCGCTGAAG	58	561	Luo et al., 2011
*bla*_CTX−M−8∕25_ group	F: AACRCRCAGACGCTCTAC^a^ R: TCGAGCCGGAASGTGTYAT^*a*^	55	326	Luo et al., 2011
*qnrA*	F: TCAGCAAGAGGATTTCTCA R: GGCAGCACTATTACTCCCA	58	627	Robicsek et al., 2005
*qnrB*	F: GATCGTGAAAGCCAGAAAGC R: ACGATGCCTGGTAGTTGTCC	53	469	Gay et al., 2006
*qnrS*	F: ACGACATTCGTCAACTGCAA R: TAAATTGGCACCCTGTAGGC	53	417	Gay et al., 2006
*qnrC*	F: GGGTTGTACATTTATTGAATC R: TCCACTTTACGAGGTTCT	52	447	Wang et al., 2009
*qnrD*	F: CGAGATCAATTTACGGGGAATA R: AACAAGCTGAAGCGCCTG	52	582	Cavaco et al., 2009
*aac(6')-Ib*	F: TTGCGATGCTCTATGAGTGGCTA R: CTCGAATGCCTGGCGTGTTT	55	482	Park et al., 2006
*qepA*	F: AACTGCTTGAGCCCGTAGAT R: GTCTACGCCATGGACCTCAC	55	596	This study
*oqxA*	F: GCGTCTCGGGATACATTGAT R: GGCGAGGTTTTGATAGTGGA	56	482	This study
*oqxB*	F: CTGGGCTTCTCGCTGAATAC R: CAGGTACACCGCAAACACTG	55	498	This study
*intI1*	F: CCTCCCGCACGATGATC R: TCCACGCATCGTCAGGC	54	280	Dillon et al., 2005
*intI2*	F: AAATCTTTAACCCGCAAACGC R: ATGTCTAACAGTCCATTTTTAAATTCTA	54	439	Dillon et al., 2005
*intI3*	F: AGTGGGTGGCGAATGAGTG R: TGTTCTTGTATCGGCAGGTG	54	599	Dillon et al., 2005
*IntI1* variable region	F: TCATGGCTTGTTATGACTGT R: GTAGGGCTTATTATGCACGC	56	Variable	White et al., 2000

a *Y = T or C; R = A or G; S = G or C; D = A or G or T*.

*F, forward; R, reverse*.

### Integrons analysis and characterization of inserted gene cassettes

PCR was performed to check the presence of class 1, 2, and 3 integrons in all 198 ESBL-positive *E. coli* isolates using primers specific for the integron integrase genes *intI1, intI2*, and *intI3* listed in Table 2 (Dillon et al., 2005). Strains that was positive for *intI1* gene were subsequently subjected to PCR for amplification of the variable region of class 1 integrons using primer listed in Table 2 (White et al., 2000). The PCR products of class 1 integrons variable regions with identical amplicon lengths were analyzed by restriction fragment length polymorphism (RFLP) using *HinfI* (TaKaRa, Japan) according to the method of previous study (Gu et al., 2008). PCR products with identical RFLP profiles were regarded as the same gene cassette array. PCR products with different gene cassette arrays were sequenced and analyzed.

### Phylogenetic genotyping

Phylogenetic grouping of the *E. coli* isolates was determined using a triplex PCR for the genes *chuA, yjaA*, and TspE4C2 as described previously (Clermont et al., 2000).

### Statistical analysis

Frequency of antimicrobial resistance profiles between ESBL-positive and ESBL-negative *E. coli* isolates were compared using the Pearson Chi-square test with the software SPSS 17.0, and the level of significance was set at *p* < 0.05.

## Results

### Bacterial strains and antimicrobial susceptibility testing

A total of 458 *E. coli* isolates were recovered from the 589 fecal samples (Table 1). Of which, 43.2% (*n* = 198) were suspected to be ESBL producers using DDST. The results of the *in vitro* antimicrobial susceptibility testing of all *E. coli* isolates are shown in Table 3. High resistance rates (>90%) were discovered among both ESBL-positive and ESBL-negative *E. coli* isolates for ampicillin, trimethoprim-sulphamethoxazole, nalidixic acid, and tetracycline. Interestingly, ESBL-positive isolates demonstrated remarkably higher resistance rates to ampicillin, ceftiofur, cefotaxime, amikacin, gentamicin, florfenicol, enrofloxacin, and ciprofloxacin than ESBL-negative isolates (*p* < 0.05). All of the isolates were resistant to at least one antimicrobial agents tested and 96.1% (*n* = 440) of the isolates were resistant to three or more classes of antimicrobial thus defined as multi-drug resistant (MDR). With regard to MDR profiles, all isolates grouped in to 65 resistance phenotypes, 92.8% isolates were resistant to more than five, 62.9% were resistant to more than 9, and 34 (7.4%) were resistant to 12 antimicrobial agents. The predominant multi-resistance profile was ampicillin/tetracycline/chloramphenicol/nalidixic acid/enrofloxacin/trimethoprim-sulphamethoxazole, which accounted for 39.1% (179/458) of all the isolates.

**Table 3:** **Antimicrobial susceptibility of ***E. coli*** strains**.

**Antimicrobial agents**	**Number (%) of resistant isolates (*n* = 458)**	**ESBL-positive** ***E. coli*** **isolates (*****n*** = 198**)**				**ESBL-negative** ***E. coli*** **isolates (*****n*** = 260**)**				***P*-value**
		**MIC (μg/ml)**			**Number (%) of resistant isolates**	**MIC (μg/ml)**			**Number (%) of resistant isolates**	
		**Range**	**MIC_50_**	**MIC_90_**		**Range**	**MIC_50_**	**MIC_90_**
Ampicillin	443 (96.7)	1->256	256	>256	198 (100)	0.5->256	16	256	245 (94.2)	< 0.001
Ceftiofur	200 (43.7)	2->256	128	>256	195 (98.5)	≤ 0.06-32	0.5	16	5 (1.9)	< 0.001
Cefotaxime	192 (41.9)	2->256	128	>256	189 (95.5)	≤ 0.06-4	0.5	2	2 (0.8)	< 0.001
Amikacin	150 (32.8)	0.5->256	64	>256	120 (60.6)	0.25-128	16	64	30 (11.5)	< 0.001
Gentamicin	332 (72.5)	1->256	128	>256	165 (83.3)	0.5->256	64	256	167 (64.2)	< 0.001
Tetracycline	430 (93.9)	2->256	256	>256	183 (92.4)	1->256	256	>256	247 (95)	0.254
Chloramphenicol	388 (84.7)	0.5->256	128	256	172 (86.9)	0.5->256	128	>256	216 (83.1)	0.264
Florfenicol	381 (83.2)	4->256	256	>256	175 (88.4)	1->256	128	>256	206 (79.2)	< 0.01
Nalidixic acid	446 (97.4)	4->256	256	>256	192 (97)	1->256	128	>256	254 (97.7)	0.631
Enrofloxacin	379 (82.8)	0.5-256	128	256	179 (90.4)	0.25-512	64	128	200 (76.9)	< 0.001
Ciprofloxacin	355 (77.5)	0.5-256	64	128	182 (91.9)	0.25-256	32	128	173 (66.5)	< 0.001
Trimethoprim/-sulfamethoxazole	438 (95.6)	0.5/9.5-64/1216	16/304	64/1216	193 (97.5)	0.5/9.5-64/1216	16/304	32/608	245 (94.2)	0.0924
Colistin	0 (0)	≤ 0.06-2	0.5	1	0 (0)	≤ 0.06-1	0.5	0.5	0	NS

*NS, no significant*.

### Characterization of ESBL and PMQR genes

PCR was performed on 198 ESBL-positive *E. coli* to determine the presence of ESBL and PMQR genes. 98.5% (195/198) of the ESBL-positive *E. coli* isolates harbored one or more of the genes from the three families of TEM, SHV, and CTX-M, whereas the remaining three isolates failed to show the presence of such genes, suggesting that these three isolates perhaps carry other ESBL genes, which will require further studies. The distribution of ESBL genotype among the 198 ESBL-positive *E. coli* isolates was shown in Table 4. 96.5% (191/198) of the isolates were detected to harbor one or two *bla*_CTX−M_ genes, and *bla*_CTX−M−1_ or *bla*_CTX−M−9_ positive groups were detected at 36.7% (70/191) and 60.2% (115/191), respectively; The *bla*_CTX−M−1_ and *bla*_CTX−M−9_ double-positive group accounted for 3.1% (6/191). None of the isolates contained the *bla*_CTX−M−2_, *bla*_CTX−M−8_, and *bla*_CTX−M−25_ genes. In the *bla*_CTX−M−1_ group, *bla*_CTX−M−55_, and *bla*_CTX−M−15_ were the major genotypes, accounting for 52.6% (40/76) and 28.9% (22/76), respectively; the remaining genotypes included *bla*_CTX−M−3_ (4), *bla*_CTX−M−64_ (4), and *bla*_CTX−M−123_ (6). In the *bla*_CTX−M−9_ group, *bla*_CTX−M−14_ was the major genotype, accounting for 58.7% (71/121); the remaining included *bla*_CTX−M−27_ (15), *bla*_CTX−M−65_ (28), *bla*_CTX−M−104_ (4), and *bla*_CTX−M−125_ (3). More than one type of CTX-M gene was identified in six isolates and their combinations were CTX-M-14 and CTX-M-3 in one isolate, CTX-M-14 and CTX-M-55 in three isolates, and CTX-M-14 and CTX-M-15 in two isolates. 75.3% (149/198) isolates were found to be positive for *bla*_TEM_, except four isolates were found to be *bla*_TEM−52_ by sequencing, the other 145 isolates were positive for *bla*_TEM−1_, and all of the *bla*_TEM_-positive isolates simultaneously carried *bla*_CTX−M_ genes. None of the isolates contained *bla*_SHV_ gene.

**Table 4:** **Distribution of ESBL genotype among ESBL-positive ***E. coli*** isolates**.

**ESBL genotype**	**NO. of isolates**
CTX-M	191
CTX-M-1 group	70
CTX-M-55	37
CTX-M-15	20
CTX-M-123	6
CTX-M-64	4
CTX-M-3	3
CTX-M-9 group	115
CTX-M-14	65
CTX-M-65	28
CTX-M-27	15
CTX-M-104	4
CTX-M-125	3
CTX-M-1+9 groups	6
CTX-M-14+CTX-M-55	3
CTX-M-14+CTX-M-15	2
CTX-M-14+CTX-M-3	1
TEM	149
TEM-1	145
TEM-52	4
SHV	0

The distribution of PMQR-encoding genes among the 198 ESBL-positive *E. coli* isolates was shown in Table 5. 87.4% (173/198) of the ESBL-producing *E. coli* isolates were found to harbor at least one PMQR gene, with *oqxAB, qnrS, qnrB, qepA*, and *aac(6*′*)-Ib-cr* being detected alone or in combination in 125 (63.1%), 114 (57.6%), 26 (13.1%), 24 (12.1%), and 45 (22.7%) strains, respectively. *qnrA, qnrC*, and *qnrD* were not found in this study. *oqxAB* and *qnrS* were the two most common PMQR genes. Among the 173 PMQR-positive isolates, 105 (60.7%) isolates were positive for more than one PMQR genes, and combinations of *oqxAB*+*qnrS* (*n* = 42) was the most common combination type, followed by *oqxAB+qnrS+aac(6*′*)-Ib-cr* (*n* = 16). Twelve other combination types of PMQR were also found in this study. Sixty-three isolates had two co-existing PMQR genes, 29 isolates harbored three PMQR genes, nine isolates harbored four PMQR genes, and three isolates harbored five PMQR genes. It is interesting to note that *oqxAB, qnrB, qnrS, qepA*, and *aac(6*′*)-Ib-cr* coexisted in three isolates. To our knowledge this is the first time to report one isolate carried six PMQR genes. Among the 125 *oqxAB*-positive isolates, 52 and 24 isolates harbored *bla*_CTX−M−14_ and *bla*_CTX−M−55_ genes, respectively. In the 110 *qnrS* positive isolates, 37 and 30 isolates harbored *bla*_CTX−M−14_ and *bla*_CTX−M−55_ genes, respectively. *qnrS, oqxAB*, and *qnrS+oqxAB* were also detected in three non-ESBL genes isolates respectively.

**Table 5:** **Distribution of PMQR genes among ESBL-positive ***E. coli*** isolates**.

**PMQR gene**	**ESBL genes (No. of isolates)**	**Total**
*oqxAB*	CTX-M-14 (17) CTX-M-27 (2) CTX-M-65 (11) TEM-52 (2)	32
*qnrS*	CTX-M-14 (3) CTX-M-15 (7) CTX-M-27 (3) CTX-M-55 (13) CTX-M-65 (3) CTX-M-123 (1)	30
*aac(6′)-Ib-cr*	CTX-M-27 (2) CTX-M-65 (1) CTX-M-104 (1)	4
*oqxAB*+*qnrS*	CTX-M-14 (15) CTX-M-15 (6) CTX-M-55 (10) CTX-M-65 (2) CTX-M-123 (1) CTX-M-14+CTX-M-3 (1) CTX-M-14+CTX-M-55 (3) CTX-M-14+CTX-M-15 (2) TEM-52 (1)	41
*qnrB*+*qepA*	CTX-M-15 (1) CTX-M-55 (1) CTX-M-64 (2)	4
*qnrS*+*qepA*	CTX-M-14 (3) CTX-M-104 (1) CTX-M-125 (1)	5
*qnrB*+*aac(6′)-Ib-cr*	CTX-M-55 (2) CTX-M-64 (2)	4
*oqxAB*+*aac(6′)-Ib-cr*	CTX-M-15 (3) CTX-M-55 (4) CTX-M-123 (2)	9
*oqxAB*+*qnrB*+*qnrS*	CTX-M-65 (1)	1
*oqxAB*+*qnrB*+*qepA*	CTX-M-14 (2) CTX-M-65 (1)	3
*oqxAB*+*qnrS*+*qepA*	CTX-M-27 (3) CTX-M-55 (2) CTX-M-125 (2)	7
*oqxAB*+*qnrB*+*aac(6′)-Ib-cr*	CTX-M-14 (2)	2
*oqxAB*+*qnrS+aac(6′)-Ib-cr*	CTX-M-14 (6) CTX-M-15 (3) CTX-M-27 (1) CTX-M-55 (2) CTX-M-104 (1) CTX-M-123 (2)TEM-52 (1)	16
*oqxAB*+*qnrB*+*qnrS*+*qepA*	CTX-M-14 (2)	2
*oqxAB*+*qnrB*+*qnrS*+*aac(6′)-Ib-cr*	CTX-M-14 (2) CTX-M-27 (2) CTX-M-65 (2) CTX-M-104 (1)	7
*oqxAB*+*qnrB*+*qnrS*+*qepA*+*aac(6′)-Ib-cr*	CTX-M-55 (3)	3

### Integrons and gene cassettes

The class 1 integron integrase gene *intI*1 was detected in 78.3% (155/198) ESBL-positive *E. coli* isolates. *intI2* and *intI3* genes were not detected in any of the isolates. The variable regions of class 1 integron was amplified using primers complementary to the conserved regions flanking the inserted resistance genes. One hundred and ten of the 155 *intI1*-positive *E. coli* isolates were positive for the variable region and the inserted gene cassette sizes varied in size from 0.75 to 1.9 kb. BLAST analysis of sequenced products of variable region of class 1 demonstrated the presence of eight different cassette combinations for 10 different genes. These genes encode for the resistance to trimethoprim (*dfrA1, dfrA5, dfrA7, dfrA12, dfrA17*, and *dfrA27*) and aminoglycosides (*aadA1, aadA2, aadA5*, and *aad22*). Out of 110 *intI1*-positive isolates 57 carried *dfrA17-aadA5* gene cassette; 28 harbored *dfrA12-orfF-aadA2* gene cassette; 12 harbored *dfrA1-aadA1* gene cassette. The sequences of the *dfrA1*-*aadA1, dfrA17*-*aadA5*, and *dfrA12*-*orfF*-*aadA2* gene cassettes have been deposited in the GenBank database under the accession numbers KT692980, KT692981, and KT692982, respectively. The distribution of integron among ESBL-positive *E. coli* was shown in Table 6.

**Table 6:** **Distribution of gene cassette arrays found in class 1 integrons among ESBL-positive ***E. coli*** isolates**.

**Gene cassette arrays**	**Amplicon size (kb)**	**ESBL genes (No. of isolates)**	**Total**
*dfrA5*	0.75	CTX-M-3 (1) CTX-M-14(2) CTX-M-55 (1)	4
*dfrA7*	0.75	CTX-M-27 (1) CTX-M-104 (1) CTX-M-123 (1)	3
*dfrA27*	0.75	CTX-M-15 (1)	1
*aadA1*	1.0	CTX-M-14 (1) CTX-M-15 (1)	2
*aadA22*	1.0	CTX-M-65 (3)	3
*dfrA1-aadA1*	1.6	CTX-M-14 (3) CTX-M-55 (5) CTX-M-64 (2) CTX-M-14+CTX-M-55 (2)	12
*dfrA17-aadA5*	1.7	CTX-M-14 (32) CTX-M-15 (6) CTX-M-27 (2)CTX-M-55 (15) CTX-M-123 (2)	57
*dfrA12-orfF-aadA2*	1.9	CTX-M-14 (10) CTX-M-15 (12) CTX-M-27(2) CTX-M-65 (4)	28

### Phylogenetic groups

All ESBL-positive isolates were allocated to one of the four phylogenetic groups A, B1, B2, and D. The results showed that 53.5% (106/198) of these isolates belonged to group A, 24.2% (48/198) to group D, and 21.2% (42/198) to group B1. Only two strains were found in group B2.

## Discussion

Over the past few years, with the development of an intensive and large-scale pig industry in China, the occurrence and epidemic of porcine diseases have taken on a lot of new characteristics (Liu et al., 2014). In particular, PWD caused by pathogenic *E. coli* is one of the most serious threat for the swine industry. During PWD outbreaks in pig farms, it was certainly thought that antimicrobial therapy would be an important control measure (Amezcua et al., 2002). However, commonly used animal antimicrobial agents did not appear to be effective for curing PWD (Moredo et al., 2015). Disturbingly, only third-generation cephalosporins such as ceftiofur and cefquinome are effective for controlling PWD (Hornish and Kotarski, 2002). The frequent use of third-generation cephalosporins as therapy or feed additives in pigs has selected high antimicrobial resistance in bacteria, which is becoming a serious issues in pig industry (Lei et al., 2010). All *E. coli* isolates in the current study were tested for their susceptibility to 13 antimicrobial agents. Similar to previous studies (Smith et al., 2010), we found a very high frequency of resistance to most of the conventional antimicrobial agents such as gentamicin, tetracycline, chloramphenicol, and trimethoprim-sulphamethoxazole normally used to treat PWD infections (Luppi et al., 2015). Certainly, these antimicrobial agents could not be the good choice to treat piglets with PWD in these farms. The remarkable finding in the present study is that 92.8% isolates were resistant to more than five antimicrobial agents, and 62.9% isolates were resistant to more than nine antimicrobial agents, which is much higher than our previous studies (Zhang et al., 2009). However, all isolates remained susceptible to colistin, which has been shown to be a safe and effective candidate to treat infections caused by *E. coli* in China pig farms (Wu et al., 2012). Comparing the frequency of resistance to these 13 antimicrobial agents, higher resistance rates were present in ESBL-positive strains than in negative ones, which suggested the former may acquire resistance easier than the latter.

The prevalence of ESBL-positive *E. coli* isolates in food-producing animals has been increasing worldwide (Liebana et al., 2013). In this study, 43.2% (198/458) of the *E. coli* isolates screened from PWD piglets were ESBL producers, which is higher than the detection rates (12.6%, 41/326) in *E. coli* from healthy pigs (*P* < 0.001; Zheng et al., 2012). These results suggest that ESBLs are more frequently present in isolates from sick animals compared to commensal *E. coli* from the healthy food-producing animals. The high prevalence rate of ESBL-positive *E. coli* in diarrhea piglets may be explained by the fact that the third-generation cephalosporins are used more commonly in swine for treatment of ND and PWD diseases (Lei et al., 2010; Barton, 2014). Nevertheless, this constitutes a potential public health concern since the recovered sick animals carrying ESBL resistant gene may enter the food chain. The inappropriate use and/or overuse of third-generation cephalosporins has been associated with the emergence and spread of ESBL-positive *E. coli* in pigs (Cavaco et al., 2008). For that reason, in many countries, third-generation cephalosporins have been forbidden for use in pig production, as a result of which, the occurrence of ESBL-positive *E. coli* has been significantly reduced (Agersø and Aarestrup, 2013). In china, however, third-generation cephalosporins are still approved for food-producing animals with no limitation.

CTX-M-type ESBLs have been isolated increasingly from human, companion, and food-producing animals *E. coli* isolates in different countries in the last several years (Zhao and Hu, 2013). A 2003–2012 survey in China showed that CTX-M-type ESBLs accounted for 87.1% of ESBL-positive *E. coli* strains isolated from food animals (Rao et al., 2014). CTX-M-14 remains the most abundant genotype, although the detection rate of CTX-M-55 and CTX-M-65 have shown a continuously increasing trend in recent years (Sun et al., 2010; Zheng et al., 2012). Similar findings were obtained in the present study, the proportion of CTX-M-producing *E. coli* had increased to 96.5%, and CTX-M-14 remained the most common genotype of ESBLs, followed by CTX-M-55 and CTX-M-65. In addition, we also found the *bla*_CTX−M−3_, *bla*_CTX−M−15_, *bla*_CTX−M−27_, *bla*_CTX−M−64_, *bla*_CTX−M−65_, *bla*_CTX−M−104_, *bla*_CTX−M−123_, and *bla*_CTX−M−125_ genes in these isolates, indicating a high diversity of *bla*_CTX−M_ genes in *E. coli* isolates from PWD piglets in Heilongjiang province. In the past, *bla*_TEM−52_ has only been detected in *E. coli* isolates from chicken in China (Li et al., 2010), but now it has been found in four *E. coli* isolates from piglets of different farms. To our knowledge, this is the first report of *bla*_TEM−52_ in *E. coli* from pigs in China.

Emergence of PMQR gene has been reported worldwide and is being documented in ESBL producers in recent days (Poirel et al., 2012). In the present study, we found that the detection rate of PMQR genes was 87.4% among a collection of 198 ESBL-positive *E. coli* isolates. Somewhat similar finding has also been reported in previous study in China, where 83.8% (160/191) of ESBL-positive *E. coli* from swine contained the PMQR genes (Liu et al., 2013b). However, the detection rate of PMQR among ESBL-positive *E. coli* in other countries was low, with 50% of the *E. coli* isolates from human in Tunisian and 13.6% *E. coli* isolates from human in Mexico (Silva-Sánchez et al., 2013; Ferjani et al., 2015). Over the past 10 years, PMQR determinants have emerged as an important issue. Their detection among ESBL-positive *E. coli* from human and food-producing animals has been widely investigated and different rates have been reported depending on the country, origin of isolates, and the number of *E. coli* included (Seiffert et al., 2013). The widespread dissemination of PMQR determinants may lead to the rapid development of fluoroquinolone resistance because they can confer low-level resistance to fluoroquinolone and promote the selection of high-level resistant strains with mutations on the chromosome. In the present study, 52 of the 125 *oqxAB* positive strains possessed *bla*_CTX−M−14_, and a significant association between the *bla*_CTX−M−14_ and *oqxAB* genes have been found, which validated the finding further that isolates with the *oqxAB* gene often possess a *bla*_CTX−M−14_-producing plasmid (Liu et al., 2013b). This suggests that spread of the *oqxAB* gene might occur concurrently with the *bla*_CTX−M−14_ gene. Notably, the *bla*_CTX−M−55_, *qnrB, qnrS, aac(6*′*)-Ib-cr, qepA*, and *oqxAB* genes were found to be carried together in three isolates. Only a few previous studies have found bacteria harboring more than three PMQR genes (Yang et al., 2014). Whether multiple PMQR determinants have an additive effect on the MIC of fluoroquinolones is unknown and warrants further investigation.

Integrons are important genetic elements responsible for carriage and spread of antibiotic resistant determinants including ESBL enzymes. ESBL genes located on integrons-like structures are being increasingly reported worldwide (Bonnet, 2004). Hence, we examined the presence of class 1, class 2, and class 3 integrons. In this study, 78.3% (155/198) of ESBL-positive *E. coli* isolates harbored class 1 integron while none of the isolates contained class 2 and class 3 integron as found in previous studies (Sun et al., 2013). Sequence analysis of the amplified variable region of class 1 integron revealed that most of the integrons possessed *dfrA* and *aadA* gene cassettes conferring resistance to trimethoprim and streptomycin as reported by several researchers previously (Kar et al., 2015). This kind of gene cassette arrangement may be responsible for emergence of multiple drug resistance among ESBL producers isolated in this study. However, further study is needed to investigate the association between class 1 integron production and dissemination of ESBL type genes in *E. coli* from China.

Several authors have analyzed the distribution of the main phylogenetic groups among *E. coli* strains isolated from human and animals. We showed that *E. coli* clinical isolates from PWD piglets carrying ESBLs, collected over 3 years, belonged mainly to phylogroup A and, to a lesser extent, to phylogroups D and B1, whereas B2 strains were very little. This is in agreement with other studies on pathogenic *E. coli* of pig origin (Wang et al., 2010; Abraham et al., 2014).

In our study we have detected a high prevalence of ESBL-positive *E. coli* isolated from PWD piglets. Future studies will be performed with the ESBL-positive isolates to find possible association among serotypes, virulence factors, resistance mechanisms, and transmission mechanisms that will be useful for epidemiological research.

In conclusion, in this study a high prevalence of ESBL-positive *E. coli* has been observed in fecal samples of PWD piglets collected from Heilongjiang province, and the high prevalence of PMQR genes and integrons were also widely detected among the ESBL-positive *E. coli* strains. The *bla*_CTX−M−14_ was found to be the dominant ESBL-encoding gene in this study, while *oqxAB* and *qnrS* were the dominant PMQR genes in the ESBL-positive *E. coli*. This is the first report of TEM-52 ESBL in bacterial isolates from pig in China. To our knowledge, this is also the first description of the coexistence of the *qnrB, qnrS, aac(6*′*)-Ib-cr, qepA*, and *oqxAB* genes in one *E. coli* strain. More studies should be carried out in the future in order to ensure that the six PMQR genes were located on the same plasmid or not.

### Conflict of interest statement

The authors declare that the research was conducted in the absence of any commercial or financial relationships that could be construed as a potential conflict of interest.
